# Factor XIII deficiency in a neonate presenting as subpial haemorrhage

**DOI:** 10.4102/sajr.v26i1.2344

**Published:** 2022-05-20

**Authors:** Monish G. Karthikeyan, Poojitha Ronda, Prabhu C. Sugumaran

**Affiliations:** 1 Department of Radiodiagnosis, Mahatma Gandhi Medical College and Research Institute, Pondicherry, India

**Keywords:** intracranial haemorrhage, subpial haemorrhage, term neonate, yin-yang sign Factor XIII, CT, MRI

## Abstract

Subpial haemorrhage is a rare cause of seizures in term neonates. A 3-day-old male infant, born at term with no history of perinatal hypoxia, presented with seizures and unremarkable physical examination in the interictal state. Imaging demonstrated left temporal subpial haemorrhage with the classic ‘yin-yang sign’ on MRI. The patient was subsequently diagnosed with factor XIII deficiency. Follow-up at 6 months and 12 months revealed encephalomalacia in the previous haemorrhagic areas with normal developmental milestones.

## Introduction

Haemorrhagic stroke in neonates typically results in mild-to-severe long-term neurological disabilities.^1,2^ The commonly occurring haemorrhages are usually intraparenchymal and involve the region of the germinal matrix in preterms.^1^ Extraparenchymal haemorrhages are rare and usually occur as a result of trauma but can also be non-traumatic, secondary to asphyxia, blood dyscrasias or vascular abnormalities and may even be spontaneous.^3^

Few cases of subpial haemorrhage have been described in the literature due to their relative rarity, which is estimated at approximately 4% according to pathological reports.^4,5,6,7^ Consequently, there is a relative paucity of knowledge regarding its aetiology, associated clinical manifestations and outcomes. The risk factors and pathophysiology of this entity are unique compared with other forms of extra-axial haemorrhage.

This case report describes subpial haemorrhage in the left temporal region and the typical ‘yin-yang sign’ described in the recent literature in a term neonate presenting with seizures. There was also intraparenchymal and intraventricular extension at presentation. Encephalomalacia of the previous haemorrhagic areas was evident at follow-up imaging six months later, but no breakthrough seizures or motor developmental delay was reported on follow-up after one year.

## Case history

A 3-day-old term male infant presented with seizures and lip-smacking. There was no fever nor any signs of recent trauma. The perinatal history was unremarkable, and the baby was delivered via spontaneous vaginal delivery. His older brother was 12 years old with no relevant medical history. There were two prior abortions – one spontaneously at the third month of gestation and another that was induced with mifepristone after the 45th day of amenorrhea. No identifiable cause was found for the prior spontaneous abortions.

Routine blood investigations revealed no significant abnormality with normal prothrombin time (PT), activated partial thromboplastin time (aPTT) and international normalized ratio (INR), normal sepsis screen and negative cerebrospinal fluid (CSF) culture. Imaging was requested to determine the cause of the seizures.

**FIGURE 1:**
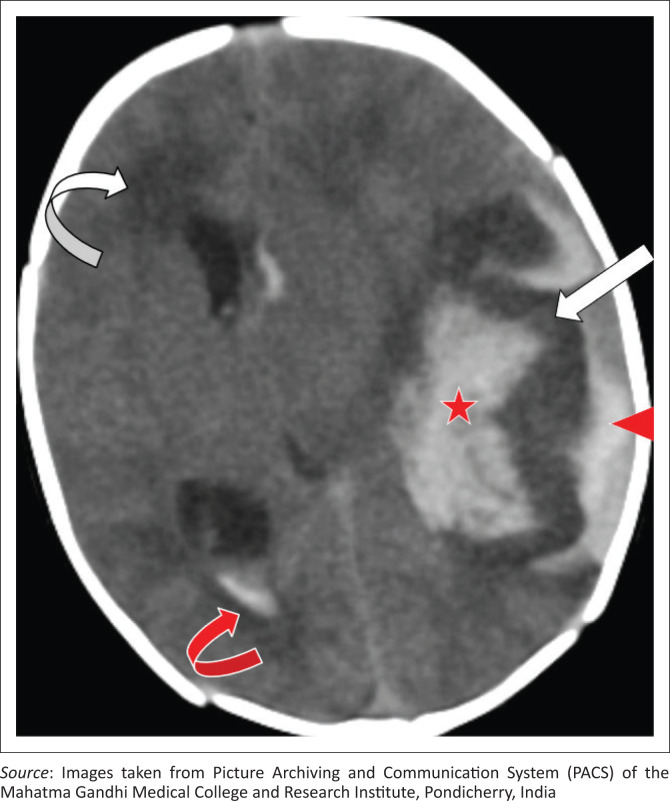
Axial CT brain. Localised extra-axial hyperdensity in the left temporal region (red arrowhead) and left temporal lobe parenchymal hyperdensity (red star) representing subpial haemorrhage with intraparenchymal extension. The cortical hypodensity (white arrow) represents ischaemia. Blood products also identified in the ventricular system (red curved arrow) with hydrocephalus and transependymal oedema (white curved arrow).

**FIGURE 2:**
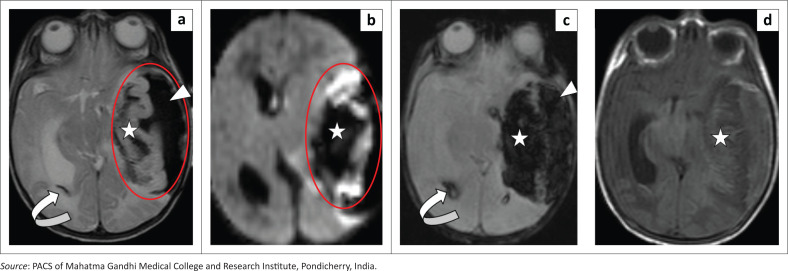
Axial MRI sequences. T2-weighted (a), diffusion-weighted imaging (DWI) (b), gradient recalled echo (GRE) (c) and T1 weighted (d) reveal left temporal T2 hypointense subpial haemorrhage with blooming on GRE. Left temporal lobe intraparenchymal extension (star), intraventricular extension and hydrocephalus noted (curved arrow). The T2-weighted and DWI sequences demonstrate the classic ‘yin-yang sign’ of subpial hypointensity and underlying cortical hyperintensity (circled in red).

An initial CT scan indicated a large hyperdense localised extra-axial haematoma in the left temporal region with intraparenchymal and intraventricular extension and mild hydrocephalus. The underlying cortex was hypodense, likely reflecting ischaemic changes (Figure 1). The MRI performed later that day confirmed the findings with the subpial haemorrhage appearing hypointense and contrasting with the underlying hyperintense cortex on T2-weighted (T2W) and diffusion-weighted imaging (DWI), resembling the ‘yin-yang sign’ (Figure 2a and b). The intraparenchymal and intraventricular extension demonstrated significant blooming on the gradient recalled echo (GRE) sequence (Figure 2c). There was also mass effect with mild midline shift to the right. Magnetic resonance venography (MRV) was unremarkable.

The patient was diagnosed with subpial haemorrhage, and therapy with oral phenobarbitone was commenced. Electroencephalogram (EEG) revealed spikes suggestive of generalised seizure activity. The baby improved symptomatically and was discharged after a week of observation with the recommendation for detailed clotting factor profile assays to exclude coagulopathies.

**FIGURE 3:**
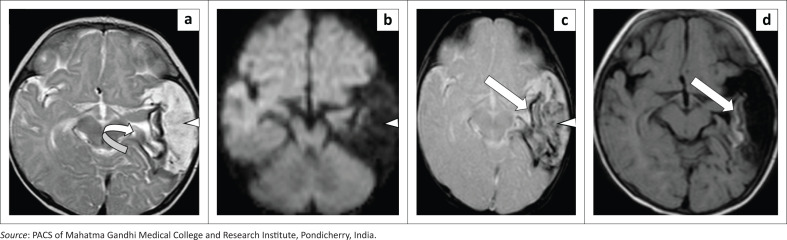
Follow-up axial MRI brain at 6 months of age. T2-weighted (a), diffusion-weighted imaging (DWI) (b), gradient recalled echo (GRE) (c) and T1 weighted (d) demonstrate resolution of the subpial haemorrhage with cystic encepahalomalacia and haemosiderin staining (arrowhead). Associated underlying temporal lobe volume loss (curved arrow). Gyriform T1 hyperintensity and blooming on GRE suggested left temporal cortical laminar necrosis with haemosiderin deposition (arrow).

Results of the clotting profile revealed factor XIII deficiency. On follow-up examination at six months of age, microcephaly was observed with the head circumference two standard deviations below normal. However, there was no significant motor neurological deficit for this age nor any breakthrough seizures. MR imaging revealed extra-axial cystic changes in the left temporal region with no new haemorrhage (Figure 3a and c). The underlying cortex demonstrated T1 hyperintensity with blooming on GRE, suggesting cortical laminar necrosis (Figure 3c and d). Therapy with oral phenobarbitone was continued, as repeat EEG showed occasional seizure-like spikes of activity.

At the age of one year, the child had started walking with support and was able to articulate words expected for his age. There were no seizures nor any evidence of motor delay. The child continues follow-up and remains seizure free.

## Discussion

Extra-axial haemorrhages are typically classified according to their location as subdural, subarachnoid or extra-dural. Subpial haemorrhage is a rare entity and can be radiologically confused with subarachnoid haemorrhage. The pia mater is the innermost, highly vascular layer of the meninges covering the entire brain and the spinal cord. It invests both the brain and spinal cord until the conus medullaris, following which it tapers and forms the bulk of the filum terminale.^8^ The pial sheath in the perivascular spaces around the intracerebral arteries is in direct continuity with them in their subarachnoid course as well.^9^ A strong trabecular meshwork is present along the vessels in the subarachnoid space but is absent in the subpial space, which may result in increased fragility of the subpial vessels.^4^

Subpial haemorrhage has been uncommonly documented and is usually ascribed to trauma or as part of child abuse – the ‘shaken baby syndrome’.^10^ The first cases of subpial haemorrhage were described in neuropathology specimens by Friede in 1975 wherein he described them as dissecting underneath the pia mater without dissecting into the subarachnoid space or the neuroparenchyma. He thought it to be a variant manifestation of respiratory distress syndrome and assumed that it began with an injury to the superficial glial cells in the molecular layer of the cortex.^7^ The same has also been described independently as ‘superficial cortical tears’ by Lindenberg in 1969, six years before Friede’s seminal work.^11^ These subcortical clefts or tears are now attributed to being a part of the secondary cascade due to impaired venous drainage and not necessarily related to traumatic events.^12^

The mechanisms by which haemorrhage occurs in the subpial space have not been definitively discovered but current theories regarding venous thrombosis have the most validity. Okudera et al. detailed the venous anatomy of the cerebral hemisphere using roentgenograms of pathological brain specimens in 1999 and described the subcortical and superficial medullary veins that run superficially and penetrate the cortex to join the pial vessels.^13^ Likely, this anatomy, combined with the absence of the trabecular mesh in the pial space, produces a potential pathomechanism by which venous injury can decompress superficial haemorrhage into the subpial space without haemorrhage in the subarachnoid space.^14^ A case report in 2015 also described subpial haematoma in the Sylvian fissure in an adult and attributed it to bleeding from small vessels in the subpial space.^15^ This may also provide the mechanism for the intraparenchymal extension that was observed in the presented case.

Subpial haemorrhages typically appear as a well-circumscribed extra-axial haemorrhage, usually seen adjacent to a gyrus. They may be bilateral, depending on the underlying pathology (such as coagulopathy), and are usually located adjacent to the cerebellum or temporal regions.^16^ Their well-localised nature and ‘yin-yang sign’ (representing the hypointense haemorrhage and hyperintense underlying cortex on T2 and diffusion-weighted MR sequences) are characteristic differentiators from other extra-axial counterparts. Additional major differentiating features are the presence of localised mass effect from the haemorrhage and ischaemic changes of the underlying cortex, which are not seen with subarachnoid or subdural haemorrhages.^17^

Roth et al. described non-traumatic subpial haemorrhage in a population of 10 patients with ages ranging from 53 years to 80 years, and observed that most of their patients recovered completely, suggesting an excellent prognosis.^4^ A cohort study of 17 patients by Cain et al. noted medullary venous congestion or thrombosis adjacent to the areas of subpial haemorrhage and suggested a venous pattern of ischaemia as the cause rather than birth trauma.^6^

Dabrowski et al. conducted the largest retrospective cohort study, to date, including 31 neonates with subpial haemorrhage. The majority were term babies (55%) with the subpial haemorrhages being commonly multifocal and if isolated, located in the temporal lobe. About 77% of cases had other haemorrhages, the most common being intraparenchymal haemorrhage. The majority of cases had no intervention but were followed up clinically and with imaging; most had some form of developmental delay on follow-up after at least 1 year.^18^ However, this was not evident in the current case study.

## Conclusion

Subpial haemorrhage is a relatively uncommon finding in clinical practice. Knowledge of its appearance, together with the recently described ‘yin-yang sign’ on MRI, will prevent misdiagnosis and exclude other extra-axial haemorrhages that might have different prognoses. The diagnosis of subpial haemorrhage should also initiate a search for background coagulopathies, which may be the underlying cause as in this case.
